# Spontaneous Cervical Intradural Disc Herniation Associated with Ossification of Posterior Longitudinal Ligament

**DOI:** 10.1155/2014/256207

**Published:** 2014-09-09

**Authors:** Dachuan Wang, Haifeng Wang, Wun-Jer Shen

**Affiliations:** ^1^Department of Orthopedics, Shandong Provincial Hospital Affiliated to Shandong University, 324 Jingwu Road, Jinan, Shandong 250021, China; ^2^Po-Cheng Orthopaedic Institute, 100 Bo-Ai 2nd Road, Kaohsiung 81357, Taiwan

## Abstract

Intradural herniation of a cervical disc is rare; less than 35 cases have been reported to date. A 52-year-old man with preexisting ossification of posterior longitudinal ligament developed severe neck pain with Lt hemiparesis while asleep. Neurological exam was consistent with Brown-Séquard syndrome. Magnetic resonance images showed a C5-6 herniated disc that was adjacent to the ossified ligament and indenting the cord. The mass was surrounded by cerebrospinal fluid signal intensity margin, and caudally the ventral dura line appears divided into two, consistent with the “Y-sign” described by Sasaji et al. Cord edema were noted. Because of preexisting canal stenosis and spinal cord at risk, a laminoplasty was performed, followed by an anterior C6 corpectomy. Spot-weld type adhesions of the posterior longitudinal ligament to the dura was noted, along with a longitudinal tear in the dura. An intradural extra-arachnoid fragment of herniated disc was removed. Clinical exam at 6 months after surgery revealed normal muscle strength but persistent mild paresthesias. It is difficult to make a definite diagnosis of intradural herniation preoperatively; however, the clinical findings and radiographic signs mentioned above are suggestive and should alert the surgeon to look for an intradural fragment.

## 1. Introduction

Intradural disc herniation is a known but rare condition, comprising less than 0.3% of all herniated discs. Over 90% of intradural disc herniations occur in the lumbar spine, less than 5% in the cervical spine [1]. Extensive digital database searches conducted by 3 previous authors on the subject and by ourselves found less than 35 reported cases in the literature.

Hsieh et al. in 2010 reported a case in which a patient who suffered from ossification of posterior longitudinal ligament (OPLL) sustained a cervical intradural disc herniation after spinal manipulation therapy [2]. We describe herein a case where a patient with OPLL developed a spontaneous cervical intradural disc herniation.

## 2. Case Presentation

The patient was a 52-year-old male with a history of posterior neck pain and subjective feeling of lower extremity weakness for 6 months. He woke up in the middle of one night with severe neck pain and Lt hemiparesis. The patient denied any recent trauma, chiropractic manipulation therapy, or even increased physical activity.

### 2.1. Examination

**Figure 1:**
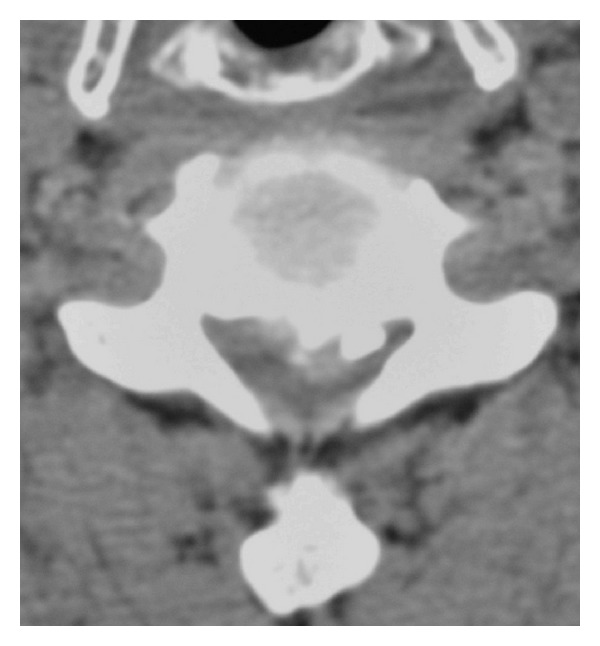
CT-scan transverse cut at the C5-6 level. Segmental type ossified posterior longitudinal ligament on the left side, with adjacent soft tissue mass protruding into the spinal canal.

**Figure 2:**
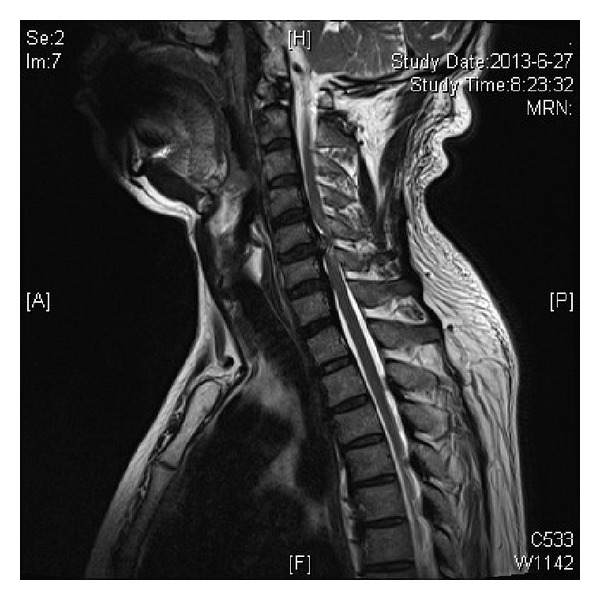
T2-weighted sagittal magnetic resonance image. A C5-6 herniated disc is seen indenting the cord. The mass is surrounded by CSF signal intensity margin, and caudally the ventral dura line appears divided into two, the so-called “Y-sign.”

**Figure 3:**
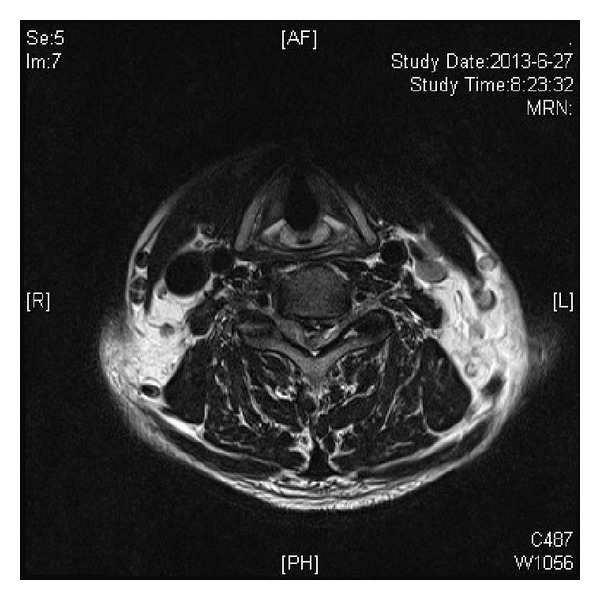
T2-weighted axial magnetic resonance image. The spinal cord is markedly compressed by a large disc herniation-like mass on the left side, with lateral displacement of the cord to the right. Zones of CSF signal intensity material (“halo”) are noted adjacent to the mass.

At the emergency room he was noted to have grade 3-4 over 5 motor hemiparaparesis on the left side and partial loss of pain and temperature sensation on the right side, consistent with a diagnosis of incomplete Brown-Séquard syndrome. A CT scan showed segmental OPLL with a large ossified mass at the C5-6 level on the left (Figure 1). T2-weighted sagittal magnetic resonance images showed a C5-6 herniated disc indenting the cord. The mass was surrounded by CSF signal intensity margin, and caudally the ventral dura line appears divided into two, reminiscent of the so-called “Y-sign” described by Sasaji et al. in 2011 [3]. High signal intensity within the cord consistent with cord edema is noted (Figure 2). T2-weighted axial MR scans showed spinal cord compression by a large disc herniation-like mass on the left side, with marked lateral displacement of the cord to the right. Again zones of CSF signal intensity material are noted adjacent to the mass (Figure 3).

### 2.2. Surgery

**Figure 4:**
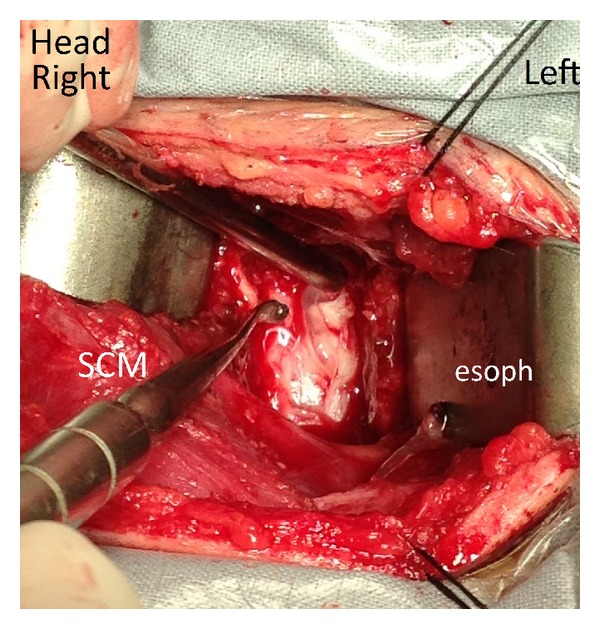
Intraoperative photograph before removal of the herniated disc fragment. Surgical approach was from the right side. There is a dural tear with a distinct preserved arachnoid layer. The disc fragment is near the tip of the metal suction. SCM = sternocleidomastoid muscle. Esoph = trachea and esophagus deep to the retractor.

**Figure 5:**
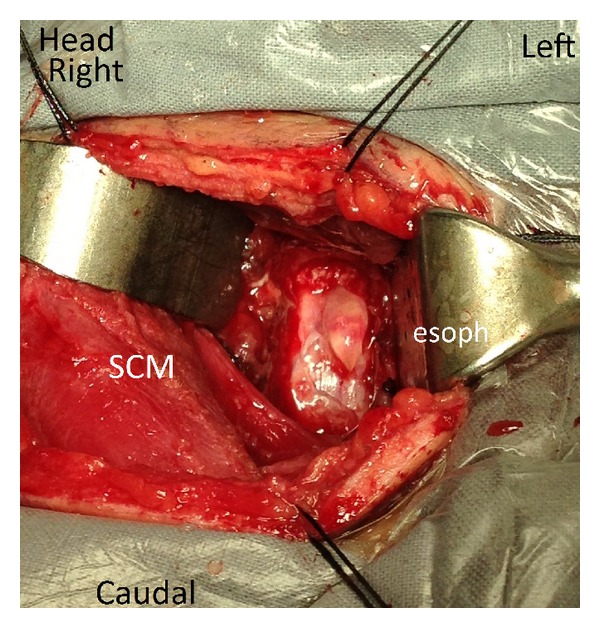
Intraoperative photograph after removal of the herniated disc fragment. Surgical approach was from the right side. The disc fragment has been removed. The near-intact arachnoid layer is seen as a bleb protruding through the dural tear. There is minimal cerebral spinal fluid leakage. SCM = sternocleidomastoid muscle. Esoph = trachea and esophagus deep to the retractor.

Because of the preexisting OPLL with spinal stenosis and the presence of cord edema consistent with a spinal cord at risk, the authors decided on a two-stage procedure. A laminoplasty was performed from C3 to C7 and secured using the Centerpiece plate fixation system (Medtronic, Minneapolis, Minnesota, USA). The posterior dura was intact and there was no CSF leakage. This was followed by an anterior C6 corpectomy. A tear was noted in the posterior longitudinal ligament (PLL) on the left side at the C5-6 disc and cranial C6 body level directly posterior to the OPLL mass. The PLL was carefully excised along with the OPLL fragment. There were spot-weld type focal adhesions between the PLL and the dura at the edges of the tear, but separation of the layers was possible. There was a 1 cm long dural defect which was plugged by an arachnoid mater bleb, with small amounts of CSF leakage. A fragment of herniated disc could be seen indenting the arachnoid (Figure 4). The disc fragment was carefully removed and the arachnoid plane was preserved, confirming that, in this case, the fragment was located in the intradural extra-arachnoid space (Figure 5). There was only slight cerebral spinal fluid leakage. The dura tear was closed and covered with Surgicel. The cervical spine was reconstructed by C5–C7 anterior interbody fusion using a titanium interbody cage filled with autogenous bone, stabilized by a titanium plate. Pathological analysis of the removed disc fragments showed fibrocartilaginous tissue and signs of degeneration.

### 2.3. Postoperative Course

**Figure 6:**
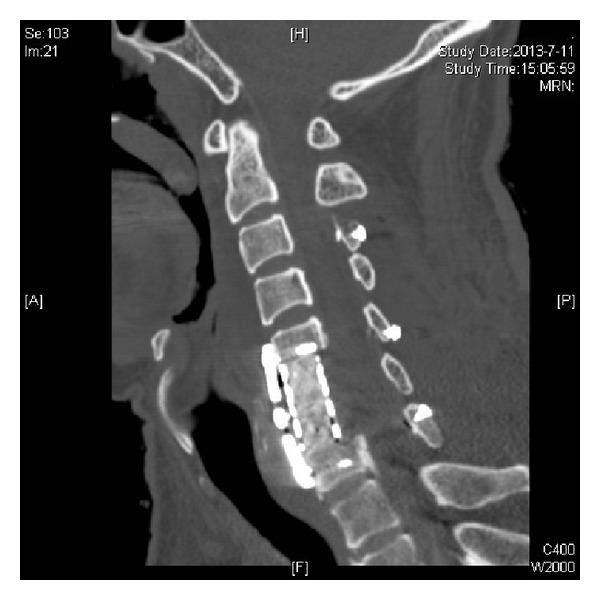
Postoperative CT-scan sagittal reconstruction. A C3–C7 laminoplasty and C6 corpectomy with reconstruction using a titanium mesh cage and plate has been done. The spinal canal is patent.

**Figure 7:**
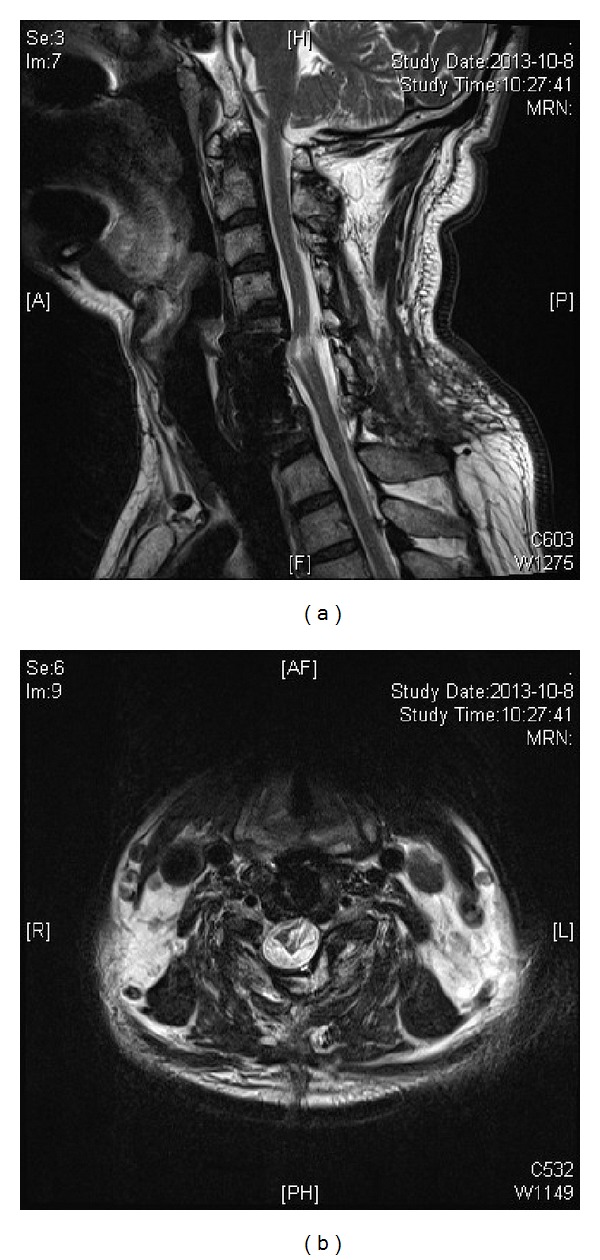
T2-weighted sagittal and axial magnetic resonance images made 3 months postoperatively. There is no stenosis, but persistent T2 high signal indicating myelomalacia is noted.

The patient had marked relief of his fulgurant pain, along with improvement of motor strength. CT scan at 2 weeks postoperatively showed good position of the implants with relief of the stenosis (Figure 6). MRI at 3 months postoperatively showed no residual cord compression; however, persistent T2 high signal indicating myelomalacia was evident (Figures 7(a) and 7(b)). Clinical exam at 6 months postoperatively revealed normal muscle strength but persistent mild paresthesias.

## 3. Discussion

Permission to present this case report was obtained from the Medical Ethics Committee of the Shandong Provincial Hospital Affiliated to Shandong University.

To the best of our knowledge, Dandy reported the first case of lumbar intradural disc herniation in 1942 [4]. The first report of intradural cervical disc herniation was published in 1959 by Marega [5]. The true incidence of this condition is not known. Tables listing previous case reports have been published by Kansal et al. (25 cases) and also by Pan et al. (27 cases) [6, 7]. Warade listed just 23 reported cases in a paper published in 2014 [8]. Our own literature search found 31 cases in 26 reports as of May 2014.

It is difficult to make a definitive diagnosis of the disease before surgery. Clinically, the most commonly reported presentation is Brown-Séquard syndrome (15 out of 32 cases), as it was in our case. Horner's syndrome has also been reported. These syndromes are by no means specific for intradural cervical disc herniation, but in our opinion they are uncommon enough that, in combination with the imaging findings discussed below, they should alert the surgeon to be on the lookout for an intradural fragment.

The pathogenesis of intradural cervical disc herniation is uncertain. Trauma is consistently listed by previous authors as a probable cause. Warade and Misra in 2014 claimed to have reported the first patient with a spontaneous cervical intradural disc herniation [8], but the results of our literature search suggested that only 7 out of 29 patients had a definite traumatic event. So while previous trauma may have played a role by inducing adhesions or by other modalities, it is not required as a precipitating event. Our patient developed a herniation in his sleep.

Yildizhan et al. in a cadaveric study published in 1991 showed that in some cases the dura mater was attached firmly to the posterior longitudinal ligament, most frequently at L4-L5, L3-L4, L5-S1, C5-C6, and C6-C7 interspaces [9]. This paper has been cited by several authors as evidence there may be a congenital anatomic predisposition.

Iwamura et al. were able to procure a gross pathological specimen from their case [10]. Hypertrophy was observed in the posterior longitudinal ligament area around the perforation. The posterior longitudinal ligament ossific lesion was palpable at the posterior portion of the C7 vertebral body. Histologic data showed fiber alignment irregularity accompanied by scattered inflammatory cell infiltration and hypertrophy of the ligament fibers.

Pan et al. noted that adhesion between ligaments and dura was often observed in case reports of intradural disc herniations [7]. In the patient reported by Hsieh et al., the hypertrophic PLL was tightly adhered to the dura mater [2]. In our case, the tear was directly posterior to the OPLL mass. There were spot-weld type focal adhesions between the PLL and the dura at the edges of the tear, especially cranially, but separation of the layers was possible. It has been theorized that degeneration of the nucleus pulposus causes secretion of increased amounts of inflammatory substances. These substances are a “foreign body” in the spinal canal, and autoimmunoreactivity will be activated [7]. We agree that biochemical factors may play an important role in the pathogenesis.

In our patient, OPLL probably caused chronic inflammation and mechanical irritation to the adjacent dura, leading to chronic scarring and adhesion of the thecal sac to the PLL. These adhesions also serve as a barrier to lateral migration of the fragment, forcing it directly dorsally through the annulus-PLL-dura layer. In Figure 5, after removal of the herniated fragment, note that the arachnoid layer is intact and formed a bleb, suggesting the forces involved were of low magnitude.

We are not aware of any reliable method to definitely identify perforation of the dural sac on MR images. In some cases one may be lucky enough to see a clear nondisplaced PLL combined with a widened subarachnoid space. Several indirect signs have been reported as being suggestive of intradural extension in the lumbar spine. Choi et al. described abrupt discontinuity of PLL and hawk-beak sign on MRI [11]. Hidalgo-Ovejero et al. focused on the presence of epidural gas on CT scan [12]. In the cervical spine, Börm and Bohnstedt call attention to a “halo” of CSF isointensity around the herniated mass on sagittal T2-weighted imaging [13].

More recently, Sasaji et al. described what they called the “Y-sign” in the lumbar spine [3]. In the case of intradural extra-arachnoid disc herniation, the arachnoid was peeled from the dura by the disc herniation. One line representing the combined dura and arachnoid was divided into two lines of the dura and the arachnoid. The branching of the ventral dural line appeared as a “Y.” We did find this sign in our case; however, we feel that it requires a measure of luck in acquiring just the right sagittal cut to see the sign.

The above mentioned signs are suggestive but not definitely predictive. We have experience with patients that had such signs on imaging, but during surgery the fragments were the usual retroligamentous epidural type. At present we feel that the diagnosis of intradural HIVD can only be confirmed by surgical exploration.

Pan et al. stated that preoperative CT scan of cervical spine is undoubtedly important and necessary in the treatment of the cervical disease, especially in exclusion of presence of OPLL, and that it was a pity that CT scan was not taken at that time in the two cases described in his article [7]. The authors agree with this. In our case the dural defect was at the location of the OPLL.

We agree with most previous authors on this subject that an anterior discectomy is the treatment of choice, as it directly addresses the problem. Whether a corpectomy is needed depends on the size and location of the extruded disc fragment. In our case the presence of OPLL and myelomalacia further prompted us to start with a laminoplasty in order to gain extra space for the cord during the anterior discectomy and fusion.
